# Genome-wide identification and expression analysis of *SMALL AUXIN UP RNA* (*SAUR*) genes in rice (*Oryza sativa*)

**DOI:** 10.1080/15592324.2024.2391658

**Published:** 2024-08-15

**Authors:** Chenhao Jia, Yujiao Shi, Hao Wang, Yaofang Zhang, Feng Luo, Zhibin Li, Yubing Tian, Xiangrui Lu, Zhongyou Pei

**Affiliations:** a Tianjin Key Laboratory of Intelligent Breeding of Major Crops, College of Agronomy & Resources and Environment, Tianjin Agricultural University, Tianjin, China; b College of Basic Sciences, Tianjin Agricultural University, Tianjin, China

**Keywords:** Rice (*Oryza sativa*), auxin, *SMALL AUXIN UP RNAs*, evolution, expression

## Abstract

*SMALL AUXIN UP RNA*s (*SAURs*), the largest family of early auxin response genes, plays crucial roles in multiple processes, including cell expansion, leaf growth and senescence, auxin transport, tropic growth and so on. Although the rice *SAUR* gene family was identified in 2006, it is necessary to identify the rice *SAUR* gene due to the imperfection of its analysis methods. In this study, a total of 60 *OsSAURs* (including two pseudogenes) distributed on 10 chromosomes were identified in rice (*Oryza sativa*). Bioinformatics tools were used to systematically analyze the physicochemical properties, subcellular localization, motif compositions, chromosomal location, gene duplication, evolutionary relationships, auxin-responsive cis-elements of the *OsSAURs*. In addition, the expression profiles obtained from microarray data analysis showed that *OsSAUR* genes had different expression patterns in different tissues and responded to auxin treatment, indicating functional differences among members of *OsSAUR* gene family. In a word, this study provides basic information for *SAUR* gene family of rice and lays a foundation for further study on the role of *SAUR* in rice growth and development.

## Introduction

Auxin plays a crucial role in various aspects of plant growth and development, including apical dominance, cell elongation, cell division, differentiation, root initiation, and tropic responses. Transcript analysis has shown that auxin exerts its effects at the molecular level by modulating the expression of several genes, including the *Auxin/Indole-3-Acetic Acid (Aux/IAA)* family, the *Gretchen Hagen 3 (GH3)* family, the *SMALL AUXIN UP RNA (SAUR)* family, aminocyclopropane-1-carboxylic acid synthase (*ACS*), glutathione-S-transferase (*GH2/4-like*), and the auxin-responsive *Gretchen Hagen3* (*GH3*) family.^1,2^ *SAUR* family members can be rapidly and strongly induced by active auxin within 2–5 minutes.^3^ Besides auxin, several studies have reported the regulation of *SAUR* gene expression by brassinosteroids (BR),^4,5^ gibberellins (GA),^6,7^ jasmonate (JA),^8^ cytokinin,^4^ and abscisic acid (ABA),^8,9^ suggesting that *SAURs* may play a role in hormone-mediated processes related to plant growth and development. However, *SAURs* are not only responsive to plant hormone induction but also to various biological and abiotic stresses, including bacterial blight,^10^ different light conditions,^4,7,11^ cold,^12,13^ drought,^13,14^ high temperature, and high salt conditions.^13,14^ Therefore, *SAUR* is involved in numerous regulatory mechanisms, indicating its diverse role in plant growth and development. *Hong Ren* and *William M. Gray* have reported that numerous developmental processes are exquisitely regulated by *SAURs*, including cell elongation,^7,15–18^ shade avoidance responses,^19,20^ high temperature-induced growth,^21^ tropic growth,^22^ apical hook development,^18^ leaf growth and senescence,^18,22,23^ root growth and development,^17,23,24^ calcium signaling,^25,26^ auxin transport,^18,24^ gravitropism and phototropism movements,^27^ and fruit abscising.^28^ Despite having a comprehensive understanding of the biological function of *SAUR* genes, the regulatory mechanisms remain poorly understood.

*SAURs* are the largest family of early auxin response genes. The first *SAUR* gene was identified from soybean.^29^ With the release of more and more plant genome information, *SAUR* families can now be described in a large number of species. There are 79 *SAURs* in Arabidopsis,^30^ 58 *SAURs* (including two pseudogenes) in rice,^31^ 71 *SAURs* in sorghum,^32^ 134 *SAURs* in potato,^1^ 99 SAURs in tomato,^13^ 91 *SAURs* in maize,^33^ 632 *SAURs* in cotton,^34^ 80 *SAURs* in apple,^35^ and 86 *SAURs* in sweet cherry.^28^ Therefore, the function of *SAUR* remains elusive, presumably due to extensive genetic redundancy.

Rice is a crucial food crop and serves as the model monocot for molecular and genetic research. Despite the identification of the *SAUR* gene family in rice as early as 2006,^31^ only a few members have been functionally characterized, including *OsSAUR39*,^24^ *OsSAUR4*,^9^ and *OsSAUR51*.^10^ There is a scarcity of reports on the *SAUR* gene family in rice. Given the continuous release of rice genome data and the limited research on the whole-genome family analysis of rice *SAUR* genes, this study aims to reanalyze the rice *SAUR* gene family. We conducted a genome-wide identification of *SAUR* genes in rice using the latest genome data to investigate their structural, genomic, gene expression features, and synteny analysis. The findings of this study will provide valuable insights for the functional characterization of *SAUR* gene family members in rice.

## Materials and methods

### Gene identification

The rice protein sequence was obtained from the Rice Genome Annotation Project (RGAP, http://rice.plantbiology.msu.edu/), while the SAUR protein sequences of Arabidopsis (78 members) were retrieved from Ensembl (http://plants.ensembl.org/index.html). The SAUR protein sequences of Zea mays and Vigna unguiculata were downloaded from NCBI (http://www.ncbi.nlm.nih.gov/).

To identify potential SAUR proteins in rice, all the SAUR amino acid sequences from *Arabidopsis*, Zea mays, and Vigna unguiculata were used as queries in local BLASTP searches (with an E-value cutoff of 1e^−5^) against the rice genome databases (RGAP). In addition, candidate *SAUR* genes of rice were further identified by conducting Hidden Markov Model (HMM) analysis. The HMM file corresponding to the SAUR domain (PF02519) was downloaded from the Pfam protein family database (E-value <1 × 10^−5^) was aligned and used to construct a rice-specific SAUR HMM using hmmbuild from the HMMER v3.1b1 suite. This new rice-specific HMM was then used to select all proteins with an E-value lower than 1. Finally, redundant sequences were manually removed. Finally, 60 proteins containing SAUR domain were identified as members of SAUR in rice using Pfam database (https://pfam.xfam.org/), SMART database (http://smart.embl-heidelberg.de/), and NCBI Conserved Domain Search database (http://www.ncbi.nlm.nih.gov/Structure/cdd/wrpsb.cgi) to confirm the conserved SAUR domain. These *OsSAUR* genes were named on the basis of their positions on pseudomolecules.

### Sequence analysis and structural characterization

The length of sequences, molecular weights, and isoelectric points of identified SAUR proteins were obtained using tools from the ExPasy website (http://web.expasy.org/protparam/). Subcellular location prediction was performed using the CELLO v2.5 server (http://cello.life.nctu.edu.tw/). Based on the aligned amino acid sequences of OsSAUR proteins (58 members, excluding 2 pseudogenes), a neighbor-joining (NJ) tree was constructed using MEGA version 7.0 with a bootstrap of 1000 replicates. The gene exon-intron structure information of *OsSAURs* was determined using their annotation information on RGAP. The MEME program (version 4.12.0) was used to identify conserved motifs in the *OsSAURs* sequences. Tbtools software^36^ was used to display the phylogenetic tree, gene structure, and conserved motifs of rice *SAUR* genes.

### Chromosomal locations and gene duplication analysis

The chromosomal localization data for these genes was obtained from RGAP. Mapchart 2.3 software was employed to generate the chromosomal location image. Additionally, the gene duplication landscape was derived using MCScanX.^37^ The putative duplicated genes were connected via connection lines.

### Phylogenetic analysis

In order to analyze phylogenetic organization of *SAUR* gene family, a combined phylogenetic tree was created with SAUR proteins from rice (58 members except 2 pseudogenes) and *Arabidopsis* (78 members), using MEGA 7.0 with NJ method and a bootstrap of 1000 replicates. And the tree is beautified with the online tool Evolview V2 (https://evolgenius.info//evolview-v2/#login).

### Upstream sequence analysis of OsSAUR genes

To investigate cis-elements in the promoter sequences of rice *SAUR* genes, we downloaded the upstream genomic DNA sequences (2000 bp) preceding the initiation codon (ATG) for each gene from the RGAP. PlantCARE, a database of plant cis-acting regulatory DNA elements (http://bioinformatics.psb.ugent.be/webtools/plantcare/html/), was used for searching auxin-responsive elements in the promoter regions of the rice *SAUR* genes.

### Microarray-based expression analysis

RiceXPro database (https://ricexpro.dna.affrc.go.jp/category-select.php) was employed to scrutinize the expression profile of *SAUR* genes in various tissues and organs at diverse developmental stages. Furthermore, the database was used to assess the expression profile of the *OsSAUR* gene in rice seedling shoots after treatment with indole-3-acetic acid (IAA) for 0, 1, 3, 6, and 12 hours.

### Plant growth conditions and stress treatments

Rice seeds are first disinfected with a 2.5% NaClO solution for 1 minute, then cleaned with 75% alcohol for 5 minutes. They are rinsed with sterile ultra-pure water for 6 times (1 min/time) and soaked in sterile water at 28°C in darkness for 2–3 days to germinate. The seedlings with white roots (selected from the same batch) are then sown in 96-well culture plates. The seedlings grown for 3 days are pre-cultured with 1/2 of the Kimura nutrient solution, and then switched to the full-strength solution after 3 days. The nutrient solution is replaced every 3 days, and the pH is adjusted to 5.5 daily. When the rice plants grow to 14 days, they are treated with 50uM IAA and sampled at 0, 3, 6, 9, and 12 hours. Each treatment is repeated three times.

### Gene expression analysis

Total *RNA* was extracted from the leaves and root of *Oryza sativa* L. seedlings using plant isolation kits (Magen, Shanghai, China, Cat. #R4312-02). Complementary DNA (cDNA) was prepared from the isolated total *RNA* using a cDNA synthesis kit with random primers (TaKaRa, Beijing, China, Cat. #RR036A). The cDNA thus obtained was used as a template for quantitative real-time PCR (qPCR) analysis using a SYBR Green real-time PCR master mix (TaKaRa, Beijing, China, Cat. #RR036A); qRT-PCR was conducted in an Applied Biosystems 7500 Fast Real-Time PCR System following the manufacturer’s instructions. Primers are designed to exclude conservative sequences. The primers used for qRT-PCR amplification are listed in Table S1. Actin1 was used as a reference gene. Gene expression in response to IAA was analyzed over time relative to that recorded at 0 h, with relative gene expression being quantified using the 2^−ΔΔCT^ method. PCR was performed in a 25 μL amplification volume consisting of 12.5 μL of 2X SYBR Green Mix, 1 μL of forward and reverse primer (10 μM), 1 μL of cDNA (100 ng/μL), ROX Reference Dye II, and 10 μL of ddH_2_O. The PCR program was as follows: 1 cycle of 95°C for 30 s, followed by 40 cycles of 95°C for 3 s, 60°C for 30 s.

### Statistical analysis

All the data in this study were analyzed using SPSS version 27.0 and the least significant difference (LSD) test. The means and standard errors were calculated, and *p* < 0.01， *p* < 0.05 was considered statistically significant in different gene expressions.

## Results

### Identification and analysis of SAUR genes in rice

In this study, 60 *OsSAURs* genes were identified (Table 1). These 60 genes were named based on their locations in rice chromosomes 1–12. The molecular weights of the rice SAUR protein ranged from 9.22 to 26.62 kDa, with *OsSAUR21* encoding the highest molecular weight and longest (252 aa) protein. *OsSAUR2* encoded the lowest molecular weight and shortest (89 aa) protein. Furthermore, the theoretical isoelectric points varied from 4.46 (OsSAUR2) to 11.55 (OsSAUR11).

**Table 1.** *SAUR* gene family in rice.

Name	Gene ID	Synonym	Chr.	Genomic Location	AA	MW (kDa)	pI	CELLO localization
OsSAUR1	LOC_Os01g06230	OsSAUR1	1	2961194 -2,961,469(+)	91	9.88	9.50	Mito(1.845)
OsSAUR2	LOC_Os01g56240	OsSAUR2	1	32390331 -32,390,805(+)	89	9.22	4.46	Chlo(2.001)
OsSAUR3	LOC_Os01g70050	OsSAUR3	1	40526591 -40,527,112(-)	173	18.06	9.35	Nucl(1.866)
OsSAUR4	LOC_Os02g05050	OsSAUR4	2	2406657 -2,407,019(+)	120	12.77	9.64	Mito(1.357)/Chlo(1.257)
OsSAUR5	LOC_Os02g05060	OsSAUR5	2	2408524 -2,409,355(-)	130	14.63	9.00	Mito(1.575)/Cyto(1.401)/Nucl(1.357)
OsSAUR6	LOC_Os02g07110	OsSAUR6	2	3655348 -3,656,185(+)	125	13.11	9.37	Nucl(1.572)
OsSAUR7	LOC_Os02g20320	OsSAUR7	2	11968305 -11,968,736(-)	143	15.31	6.00	Nucl(1.376)/Cyto(1.338)
OsSAUR8	LOC_Os02g24700	OsSAUR8	2	14333106 -14,334,014(+)	96	10.70	7.73	Mito(1.640)/Nucl(1.136)
OsSAUR9	LOC_Os02g24740	OsSAUR9	2	14355563 -14,356,170(+)	108	11.55	6.82	Mito(1.499)/Chlo(1.200)/Cyto(1.048)
OsSAUR10	LOC_Os02g30810	OsSAUR10	2	18371442 -18,372,892(-)	166	17.38	8.71	Nucl(2.052)
OsSAUR11	LOC_Os02g42990	OsSAUR11	2	25878901 -25,879,880(+)	190	21.04	11.55	Nucl(2.516)
OsSAUR12	LOC_Os02g52990	OsSAUR12	2	32418493 -32,419,361(+)	128	13.74	9.64	Nucl(2.174)
OsSAUR13	LOC_Os03g18050	OsSAUR13	3	10057658 -10,058,281(+)	207	21.92	4.99	Chlor(3.397)
OsSAUR14	LOC_Os03g25289		3	14438731 -14,439,378(-)	215	22.78	4.81	Chlo(2.900)
OsSAUR15	LOC_Os03g45800	OsSAUR14	3	25866208 -25,866,564(-)	118	12.29	7.95	Nucl(2.293)
OsSAUR16	LOC_Os03g45830	OsSAUR15	3	25884304 -25,884,624(+)	106	11.10	6.55	Nucl(2.158)
OsSAUR17	LOC_Os03g45850	OsSAUR16	3	25893200 -25,894,879(+)	134	14.14	8.69	Nucl(1.898)
OsSAUR18	LOC_Os03g45860	OsSAUR17	3	25898706 -25,899,026(+)	106	10.85	6.71	Nucl(2.463)
OsSAUR19	LOC_Os04g43740	OsSAUR18	4	25896733 -25,897,489(+)	129	14.27	6.96	Mito(1.723)/Cyto(1.297)
OsSAUR20	LOC_Os04g51890	OsSAUR20	4	30783636 -30,784,530(+)	176	18.94	9.00	Nucl(2.792)
OsSAUR21	LOC_Os04g52670	OsSAUR21	4	31349656 -31,350,722(-)	252	26.62	10.58	Mito(1.429)/Plas(1.319)
OsSAUR22	LOC_Os04g52684		4	31357557 -31,358,028(+)	100	10.28	9.21	Nucl(1.766)
OsSAUR23	LOC_Os04g56680	OsSAUR22	4	33789435 -33,790,386(-)	143	15.50	9.18	Mito(1.815)
OsSAUR24	LOC_Os04g56690	OsSAUR23	4	33804331 -33,805,473(-)	153	16.89	10.04	Nucl(2.300)
OsSAUR25	LOC_Os06g04590	OsSAUR24	6	2000167 -2,000,569(+)	119	13.26	8.95	Nucl(1.645)
OsSAUR26	LOC_Os06g45950	OsSAUR25	6	27830246 -27,831,317(-)	140	14.65	7.70	Mito(1.122)/Nucl(1.119)
OsSAUR27	LOC_Os06g45970	OsSAUR26	6	27862248 -27,862,649(+)	133	13.39	9.23	Nucl(2.211)
OsSAUR28	LOC_Os06g48850	OsSAUR27	6	29577974 -29,578,378(+)	134	14.75	8.78	Mito(1.522)/Nucl(1.302)/Cyto(1.235)
OsSAUR29	LOC_Os06g48860	OsSAUR28	6	29580155 -29,580,898(-)	140	15.11	9.55	Nucl(3.328)
OsSAUR30	LOC_Os06g50040	OsSAUR29	6	30314749 -30,315,668(-)	141	14.18	9.26	Nucl(1.919)
OsSAUR31	LOC_Os07g29310	OsSAUR30	7	17186673 -17,187,640(+)	120	12.54	10.06	Nucl(1.589)
OsSAUR32	LOC_Os08g02520	OsSAUR31	8	1039569 -1,040,187(+)	109	11.94	8.56	Chlo(1.421)/Mito(1.164)/Nucl(1.051)
OsSAUR33	LOC_Os08g02530	OsSAUR32	8	1045709 -1,046,017(+)	102	10.86	9.18	Mito(1.795)
OsSAUR34	LOC_Os08g35110	OsSAUR33	8	22136791 -22,137,879(+)	133	14.17	6.19	Mito(1.592)/Nucl(1.255)
OsSAUR35	LOC_Os08g42198	OsSAUR35	8	26694538 -26,695,159(-)	143	15.34	5.82	Cyto(1.454)/Nucl(1.069)
OsSAUR36	LOC_Os08g43700	OsSAUR36	8	27622340 -27,623,025(-)	143	15.85	8.51	Chlo(1.419)/Nucl(1.285)
OsSAUR37	LOC_Os09g26590	OsSAUR37	9	16124897 -16,125,912(+)	165	16.64	8.78	Nucl(2.383)
OsSAUR38	LOC_Os09g26610	OsSAUR38	9	16143735 -16,144,535(-)	190	20.42	4.66	Cyto(1.965)
OsSAUR39	LOC_Os09g32984		9	19691525 -19,691,941(-)	138	14.97	9.12	Nucl(1.644)
OsSAUR40	LOC_Os09g37330	OsSAUR39	9	21565736 -21,566,560(-)	171	19.20	9.26	Nucl(1.820)/Mito(1.220)
OsSAUR41	LOC_Os09g37350	OsSAUR40	9	21569466 -21,569,882(-)	138	15.22	8.15	Nucl(2.176)
OsSAUR42	LOC_Os09g37369	OsSAUR41	9	21577454 -21,578,039(+)	141	15.08	6.71	Chlo(2.521)
OsSAUR43	LOC_Os09g37380	OsSAUR42	9	21579912 -21,580,334(+)	140	15.06	7.02	Mito(1.761)/Extr(1.212)
OsSAUR44	LOC_Os09g37390	OsSAUR43	9	21582246 -21,582,833(+)	Stop codon within ORF			
OsSAUR45	LOC_Os09g37394	OsSAUR44	9	21584028 -21,584,724(+)	144	15.96	9.15	Chlo(1.473)/Mito(1.079)
OsSAUR46	LOC_Os09g37400	OsSAUR45	9	21586113 -21,586,782(+)	141	15.62	8.44	Extr1.754)/Mito(1.278)
OsSAUR47	LOC_Os09g37410	OsSAUR46	9	21590478 -21,590,900(+)	140	15.50	8.19	Mito(1.175)/Extr(1.108)
OsSAUR48	LOC_Os09g37420	OsSAUR47	9	21592239 -21,592,829(+)	110	12.55	9.30	Mito(1.487)
OsSAUR49	LOC_Os09g37430	OsSAUR48	9	21594332 -21,595,017(+)	157	17.53	8.73	Extr(1.375)/Mito(1.298)
OsSAUR50	LOC_Os09g37440	OsSAUR49	9	21596204 -21,596,638(+)	144	15.98	9.13	Mito(1.514)/Chlo(1.166)
OsSAUR51	LOC_Os09g37452	OsSAUR50	9	21598530 -21,598,726(-)	5´ end missing			
OsSAUR52	LOC_Os09g37460	OsSAUR51	9	21599723 -21,600,187(+)	144	15.62	6.06	Nucl(2.174)
OsSAUR53	LOC_Os09g37470	OsSAUR52	9	21601129 -21,601,729(-)	141	15.52	8.93	Mito(1.856)
OsSAUR54	LOC_Os09g37480	OsSAUR53	9	21602929 -21,603,542(-)	144	15.91	8.82	Chlo(1.164)/Plas(1.002)
OsSAUR55	LOC_Os09g37490	OsSAUR54	9	21604588 -21,605,172(-)	141	15.60	8.13	Extr(1.531)/Chlo(1.170)
OsSAUR56	LOC_Os09g37495		9	21606519 -21,607,126(-)	138	15.12	6.94	Mito(1.506)/Extr(1.085)/Nucl(1.073)
OsSAUR57	LOC_Os09g37500	OsSAUR55	9	21609269 -21,610,023(+)	150	15.96	8.44	Chlo(2.194)
OsSAUR58	LOC_Os10g36703	OsSAUR56	10	19631246 -19,632,264(-)	125	13.94	7.26	Nucl(1.419)/Cyto(1.106)/Mito(1.076)
OsSAUR59	LOC_Os12g41600	OsSAUR57	12	25744009 -25,745,623(-)	173	18.13	8.64	Chlo(1.819)/Nucl(1.377)
OsSAUR60	LOC_Os12g43110	OsSAUR58	12	26768220 -26,768,893(+)	130	13.49	6.07	Nucl(2.231)

Further analysis revealed that the predicted protein localization of most OsSAURs was in the nucleus, mitochondria, and chloroplasts. The remaining sequences were predicted to locate in the cytoplasm, extracellular space, and plasma membrane.

### Gene structure and motif analysis of OsSAUR

**Figure 1.**
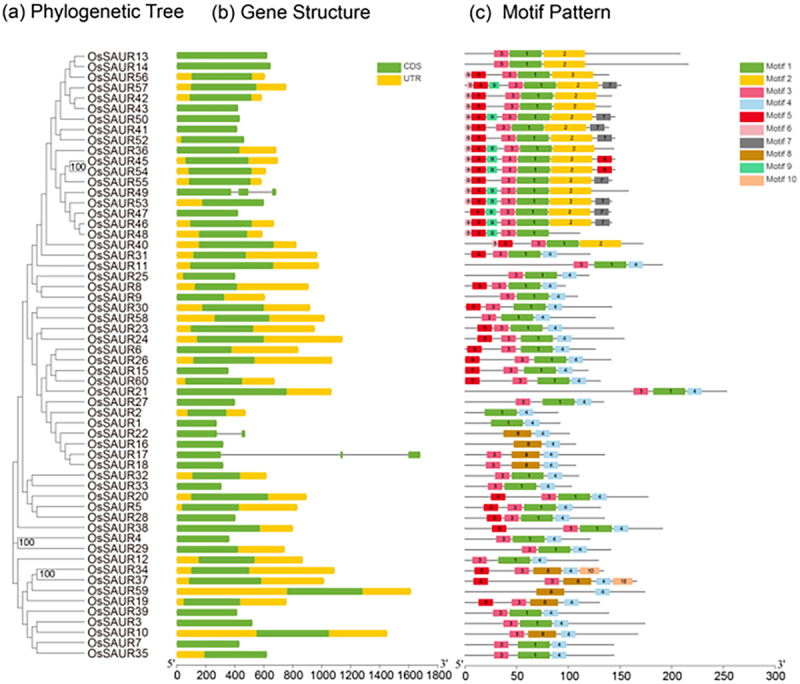
Phylogenetic relationship, gene structure and conserved motif analysis of *OsSAUR* genes. (a) Phylogenetic tree of 58 OsSAUR proteins. An unrooted neighbor-joining phylogenetic tree was constructed using MEGA7.0, based on full-length amino acid sequences of 58 OsSAUR proteins. Bootstrap testing was performed with 1000 replicates to ensure the robustness of the tree. (b) Exon/intron/intron organization of *OsSAUR* genes. Green boxes represent exons, while black lines represent introns. The upstream/downstream region of *OsSAUR* genes is indicated by yellow boxes. The length of exons can be inferred from the scale at the bottom. (c) Distributions of conserved motifs in *OsSAUR* genes. Ten putative motifs are indicated in differently colored boxes.

An unrooted NJ tree was constructed using the entire protein sequences of 58 OsSAURs (Figure 1a). In the phylogenetic tree, three sister pairs with high bootstrap values (≥99%) were identified, including OsSAUR45/OsSAUR54, OsSAUR4/OsSAUR29, and OsSAUR34/OsSAUR37. Notably, *OsSAUR* genes with a closer phylogenetic relationship generally exhibit more similar intron/exon structures and motif patterns.

**Figure 2.**
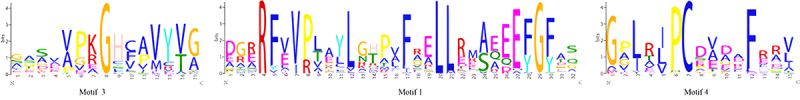
The conserved consensus motif among rice SAUR proteins according to MEME. The symbol heights indicated the relative frequency for each residue.

As depicted in Figure 1b, 55 rice *SAUR* genes lack introns, while the rest have one to two introns. *OsSAUR22* has only one intron, and *OsSAUR17* and *OsSAUR49* have two introns each. Using the MEME tool, conserved motifs in the 58 OsSAUR proteins were identified. Ten distinct motifs are depicted in Figure 1c, with details provided in Additional file 3. Almost all rice *SAUR* genes possess motifs 1, 3, and 4 (Figure 2), suggesting that they contribute to the conserved function of *SAUR* genes. In contrast, the presence of other motifs results in functional diversity among *SAUR* genes.

### Chromosomal location and duplication events of OsSAUR genes

**Figure 3.**
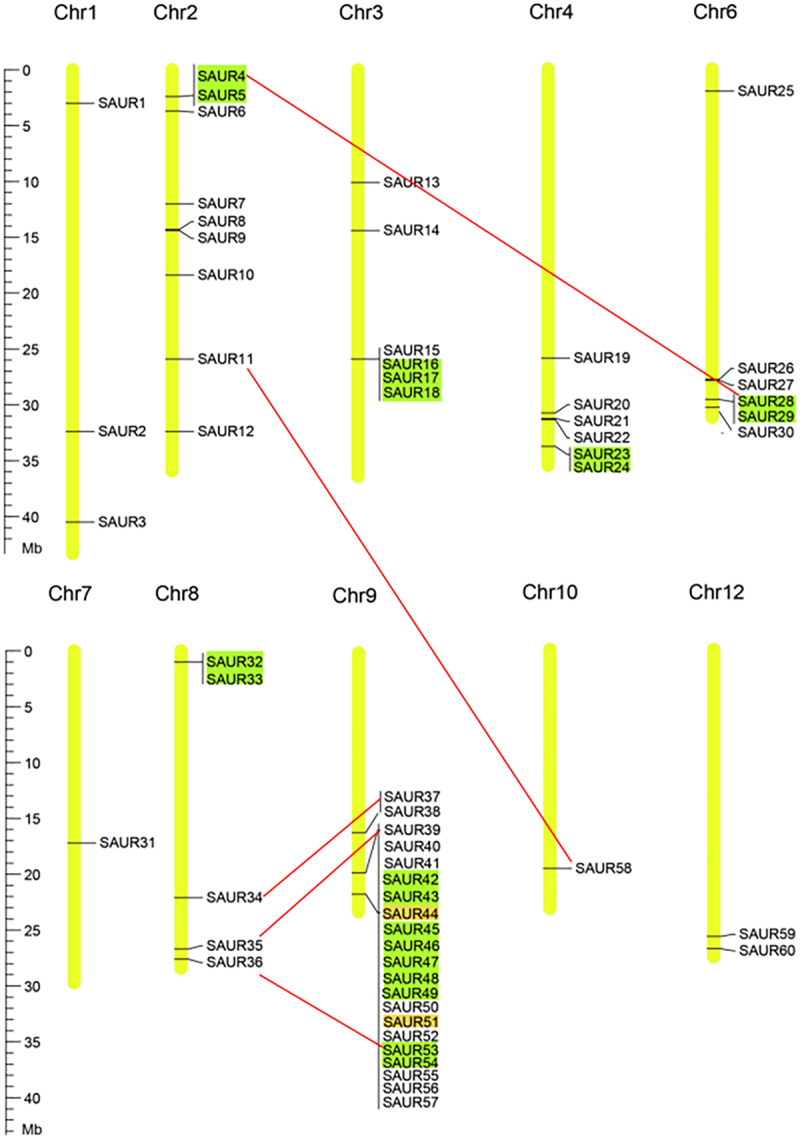
Chromosomal distribution and gene duplications of *OsSAURs*. The chromosome number is indicated at the top of each chromosome. Tandem duplicated genes are visualized as green rectangles, while segmental duplicated genes are connected by red lines. Pseudogenes are denoted by orange rectangles. The scale bar on the left indicates the length (mb) of rice chromosomes.

The 60 *OsSAUR* genes were randomly distributed across 10 rice chromosomes, except for chromosomes 5 and 6 (Figure 3). For instance, up to 21 *OsSAUR* genes were present on chromosome 9, while only one gene was found on chromosomes 7 and 10. Notably, some *OsSAUR* genes clustered at a single locus, possibly indicating gene expansion. Throughout the process of evolution, both tandem duplication and segmental duplication contributed to the formation of gene families. Therefore, we analyzed the duplication events of *OsSAUR* genes. As shown in Figure 3, 20 genes (33.3%) were confirmed to be tandem duplicated genes. Six separate pairs of tandem duplicated genes were located on chromosomes 2, 4, 6, 8, 9, and 12. One group of three tandem duplicated genes was located on chromosome 3. Additionally, five tandem duplicated genes were found on chromosome 9. According to Figure 3, five pairs of genes (*OsSAUR4/OsSAUR28*, *OsSAUR11/OsSAUR58*, *OsSAUR34/OsSAUR37*, *OsSAUR35/OsSAUR39*, *OsSAUR36/OsSAUR53*) are segmentally duplicated genes. Segmental duplication accounted for only 16.7% of the *OsSAUR* genes. It can be seen that tandem replication and segmental replication contribute to the common expansion of the *OsSAUR* family, with the former playing a leading role.

### Phylogenetic analysis of SAUR genes in rice and Arabidopsis

**Figure 4.**
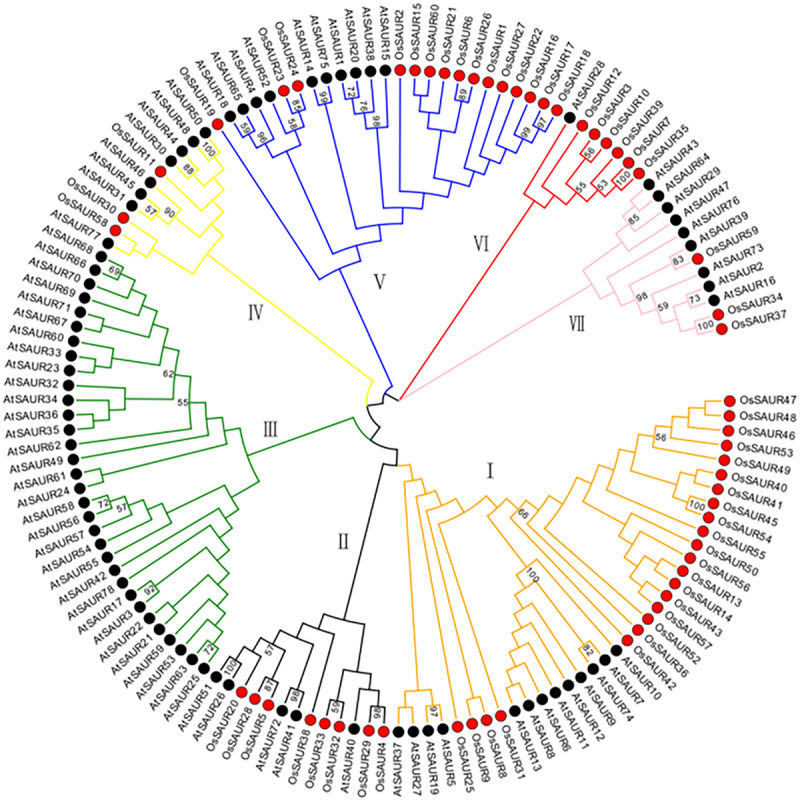
Phylogenetic tree constructed using MEGA 7.0 with NJ method and 1000 bootstrap replicates shows clustering of SAUR proteins from *Arabidopsis* (black) and *rice* (red) into 7 groups (designated as I, II, III, IV, V, VI, and VII). Bootstrap values less than 50 are not displayed in the NJ trees.

The *SAUR* gene family in rice and *Arabidopsis* can be divided into 7 groups, labeled as I to VII, based on the comprehensive phylogenetic tree (Figure 4). Groups I, III, and V are the three largest groups in the phylogeny, consisting of 36, 32, and 32 *SAUR* members, respectively. Group VI, on the other hand, contains only 7 members. In the joint phylogenetic tree, a total of 6 sister pairs (bootstrap ≥99%) were identified, including 3 pairs of *AtSAUR-AtSAUR* and 3 pairs of *OsSAUR-OsSAUR*. Notably, no sister pairs were found between dicotyledons and monocotyledons. As depicted in Figure 2, most of the groups include both species, but group III is specific to *Arabidopsis* and does not include rice. These findings suggest that a presumed gene loss event occurred between the dicot-monocot split. As expected, other groups containing several SAUR proteins from different plant species indicate that their functions remain unchanged during evolution.

### Auxin-responsive cis-elements in the promoters of OsSAUR genes

**Figure 5.**
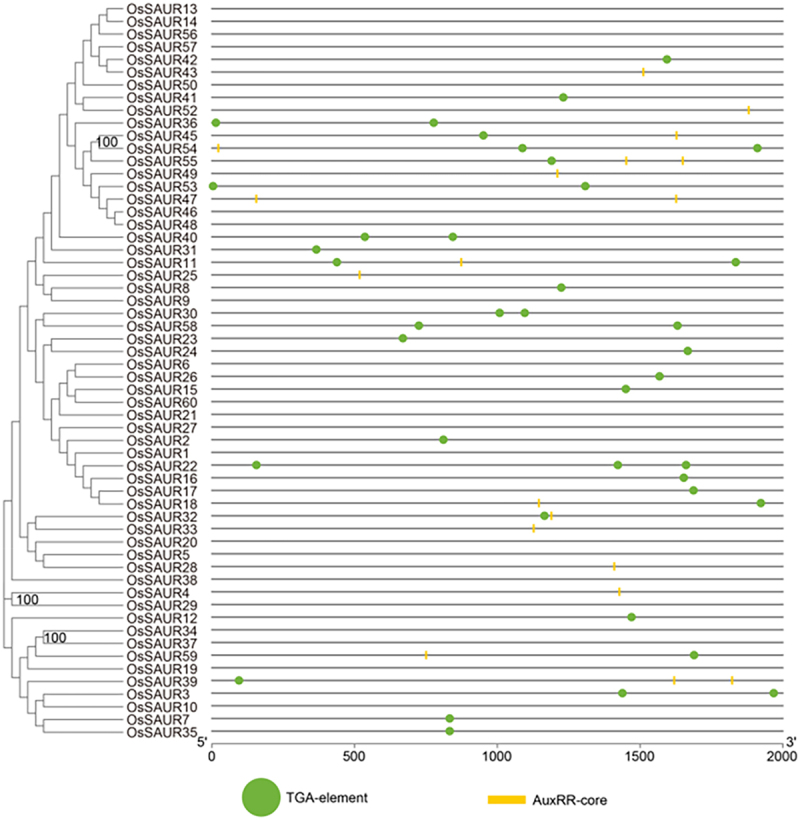
Predicted auxin-responsive cis-elements in *OsSAUR* promoters. The promoter sequences (−2000 bp) of 58 *OsSAUR* genes (excluding *OsSAUR44* and *OsSAUR51*, which lack promoter regions) were analyzed using PlantCARE. The distance upstream to the translation start site can be estimated based on the scale provided at the bottom.

To elucidate the possible regulatory mechanism of *OsSAUR* genes in exogenous auxin stimuli, we identified putative auxin-responsive cis-elements in the 2000 bp promoter regions upstream of the translation start site of the *OsSAUR* genes. Two types of auxin-responsive cis-elements, AuxRE and TGA-box, were detected by searching the promoter sequence against the PlantCARE database (Figure 5). Almost 30% of *OsSAUR* genes lack auxin responsive elements, such as *OsSAUR13*, *OsSAUR29*, and *OsSAUR34*, indicating that not all *OsSAUR* expressions are associated with auxin. Moreover, the promoters of most members are rich in a variety of cis elements, including TGACG-motif, ABRE, P-box, GARE-motif, MBSI, G-box, LTR, and MBS. The cis-element analysis indicates that *OsSAUR* genes can respond to different abiotic stresses, such as auxin, ABA, MeJA, SA, gibberellin, flavonoid biosynthetic pathways, light, cold, and drought (Table s2).

### Expression profiling of rice SAUR genes

**Figure 6.**
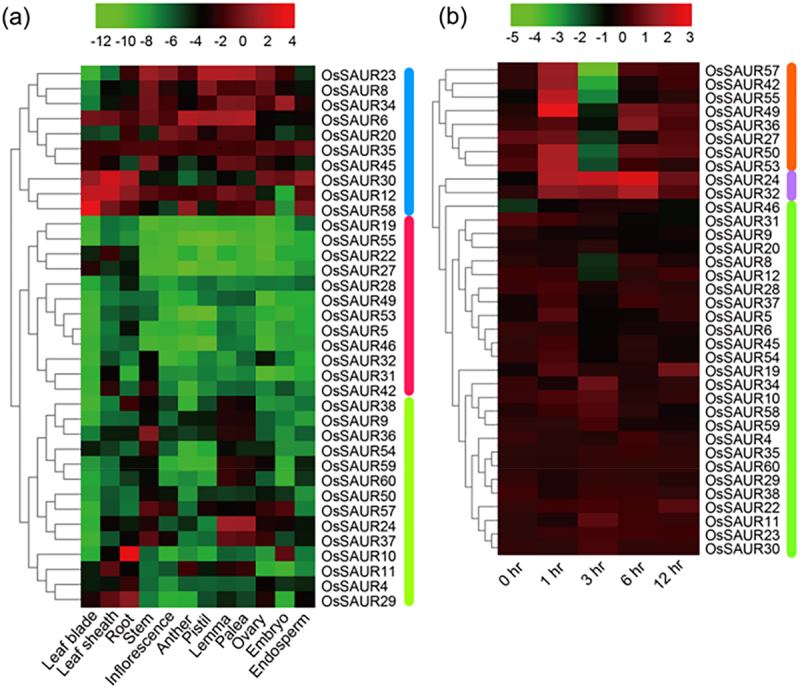
Expression profiles of rice *SAUR* genes. (a) Heatmap of *OsSAUR* gene expression in various tissues and organs. The expression profile of each gene in different tissues is displayed as normalized data (log2). (b) Expression patterns of *OsSAUR* genes in shoots of seven-day-old rice seedlings, collected at 0, 1, 3, 6, and 12 hours post-iaa treatment. The time-course expression profile of each gene is shown as the log-ratio of signal intensity (Log2Cy5/Cy3). Cy3 represents the mock treatment, while Cy5 represents the hormone treatment.

Expression profiling is crucial for understanding gene function. To predict the potential functions of *OsSAURs* in rice growth and development, transcriptomes were analyzed in various organs and tissues (including leaf blade, leaf sheath, root, stem, inflorescence, anther, pistil, lemma, palea, ovary, embryo, and endosperm) using publicly available microarray data (Figure 6a).

Only 36 *OsSAUR* transcriptomes were found in the RiceXPro database. As illustrated in Figure 6a, these 36 detected transcripts exhibit varying expression levels in different tissues, suggesting their potential involvement in diverse biological processes. Cluster analysis reveals that the tissue expression patterns of *OsSAUR* genes in rice can be categorized into three distinct types. Firstly, *OsSAUR* genes exhibit high expression in nearly all tissues, indicating that they likely contribute significantly to the overall growth and development of rice, as exemplified by *OsSAUR23*. Secondly, the expression of *OsSAUR* genes is not particularly high in most tissues, suggesting that they may not participate in rice growth and development or that their function is only activated under specific stimulation conditions, as seen with *OsSAUR19*. Thirdly, the *OsSAUR* gene is highly expressed in only a few tissues, indicating its obvious tissue specificity and involvement only in specific tissues. For instance, *OsSAUR9*, *OsSAUR24*, *OsSAUR59*, and *OsSAUR60* exhibit relatively high expression levels in the lemma and palea, suggesting these genes may play crucial roles in seed development. *OsSAUR5*, *OsSAUR10*, and *OsSAUR28* show high expression in roots, indicating they might affect the growth and development of rice roots. The specific expression of *OsSAUR11* in the leaf sheath suggests it may play a role in leaf sheath development.

To gain a deeper understanding of the regulatory mechanism of gene participation, we conducted an analysis on the expression of these 36 genes under IAA treatment (Figure 6b). The findings revealed that the expression patterns of *OsSAUR* gene in rice can be categorized into three types following auxin treatment. *OsSAUR* gene expression in response to auxin treatment varies among different genes. Some genes, such as *OsSAUR57*, show a short-term up-regulation followed by a decrease at 3 h and a subsequent up-regulation again. Others, like *OsSAUR24*, exhibit an initial up-regulation that peaks at 6 h and then decreases gradually. Finally, genes like *OsSAUR46* show no significant difference in expression levels. Overall, the response of *OsSAUR* genes to auxin is inconsistent and specific.

### Expression response of OsSAUR gene under IAA treatment

**Figure 7.**
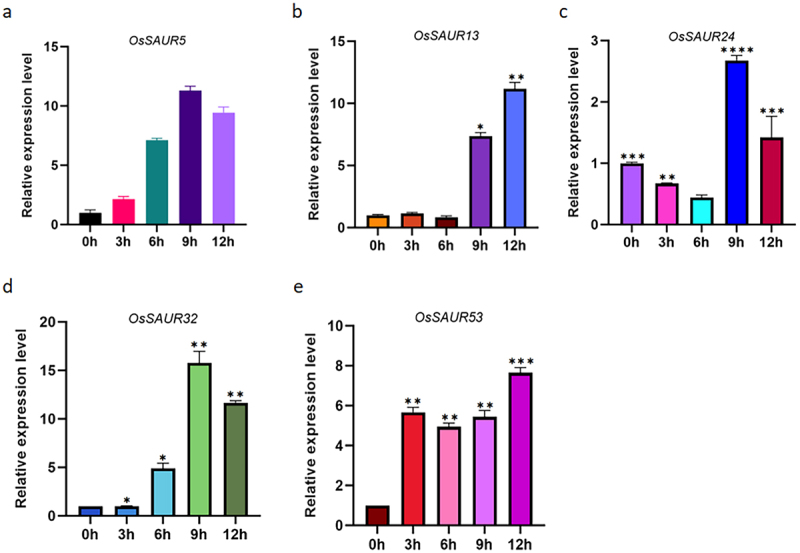
Expression of *OsSAUR5/13/24/32/53* genes under 50 μM IAA treatment (b, c, d, *p < 0.05, **p < 0.01; e, *p* < 0.05).

Additionally, to compare the induction kinetics of *SAUR* gene expression with IAA, we selected *OsSAUR5/13/24/32/53* genes from the branches of the evolutionary tree of the rice *OsSAUR* gene family for qRT-PCR analysis. All of the *OsSAUR5/13/24/32/53* genes were significantly induced under 50 μM IAA treatment (Figure 7). Among them, *OsSAUR5/32* reached the highest expression at 9 h (Figure 7a,d), while *OsSAUR13* was significantly induced starting at 9 h and continued to be induced for the following 12 h (Figure 7b). *OsSAUR24* was significantly induced at 9 h, and its expression was significantly reduced at 12 h, and its expression reached the highest expression at 9 h (Figure 7c), and it was possible that it would be continued to be induced at the following time as well. *OsSAUR53* was significantly induced at 3 hr and had high expression for a sustained period of 12 hr (Figure 7e).

## Discussion

*SMALL AUXIN UP RNAs* (*SAURs*), the largest family of early auxin response genes, play crucial roles as effector outputs of hormonal and environmental signals that regulate plant growth and development.^30^ Previous studies on the *SAUR* gene family have been conducted in various plants, including *Arabidopsis*,^2^ rice,^31^ sorghum,^32^ maize,^33,38^ cotton,^34^ and others. Although the *SAUR* gene family of rice was analyzed as early as 2006, our research on *SAUR* gene family is more comprehensive and accurate due to different analysis methods and standards. Through gene family analysis, we can examine gene structure, evolution, and function. In the current study, a search for *SAUR* genes in the rice genome led to the identification of 60 members (including 2 pseudogenes), which were designated *OsSAUR1* through *OsSAUR60* based on their chromosomal location.

Previous studies have identified 79, 71, 134, 99, and 91 *SAUR* genes in *Arabidopsis*, sorghum, potato, tomato, and maize, respectively. The genome sizes of these species are 125, 730, 844, 900, and 2300 Mb, respectively.^13,30,32,33^ The results of these studies indicate that the number of *SAUR* genes is not dependent on the genome size of the species. This finding is consistent with previous research, which showed that the rice genome, despite being larger than that of *Arabidopsis*, has a smaller number of *SAUR* genes.^39^ Gene replication events play a role in the expansion of gene families and the evolution of genomes. Tandem and segmented replication are the main models of gene replication.^40,41^

In this study, 60 *OsSAUR* genes were randomly distributed across 10 rice chromosomes, with some genes clustered. This might be due to gene duplication. Consequently, we examined *OsSAUR* gene duplication events. The findings revealed that gene replication expanded the *SAUR* gene family in rice, with tandem replication playing a significant role (Figure 3). Similar outcomes have been reported in rice, sorghum, and maize.^31,38^ Additionally, it has been noted that genes amplified by tandem replication are often involved in the response to environmental stimuli.^42,43^ In a sense, this suggests that *SAUR* genes may aid plants in adapting to environmental changes.

To some extent, genomic structure can be beneficial in predicting gene function. Genes with fewer introns are advantageous for rapid gene activation, enabling them to respond quickly to various stresses.^44^ In this study, approximately 92% of *SAUR* genes in *rice* were found to lack introns. Similar findings have been observed in other species, such as *maize* and *cotton*.^31,34,38^ These studies revealed that none of the *OsSAURs* in rice contain any introns, which differs from the results of our study. We speculate that this discrepancy may be due to variations in genomic databases and analysis methods. Previous research demonstrated that auxin can rapidly and strongly induce *SAUR* gene expression within 2–5 minute.^3^ It is possible that this is due to the fact that the majority of *SAUR* genes do not contain introns. To further investigate the conserved motif of SAUR proteins, we utilized MEME. Our analysis revealed a conserved motif of 60 amino acids in most sequences (Figure 2), which is similar to SAURs in *Arabidopsis*, tomato, potato, tobacco, sorghum, and watermelon.^13,39^ This finding suggests that *SAUR* genes share functional similarities. However, we noticed that motif 4 is absent in some *OsSAURs*, such as *OsSAUR13*, *OsSAUR14*, *OsSAUR5*, and *OsSAUR40*. We speculate that this could potentially alter the protein structure and accelerate functional differentiation.

In the early studies, *SAUR* genes of *Arabidopsis* and *rice* were categorized into two subfamilies (A and B).^31^ In this study, the full-length protein sequences of *SAUR* genes from *Arabidopsis* (78) and Rice (58) were grouped into I-VII (Figure 4). This discrepancy may be due to variations in *SAUR* members and classification criteria. It is apparent that most *OsSAURs* are clustered in groups I, II, IV-VII. This suggests that these SAUR proteins may have undergone more conservative functional evolution. However, group III only includes specific gene clusters from *Arabidopsis*, indicating distinct evolutionary patterns between *rice* and *Arabidopsis*. This species-specific expansion has been reported in rice, tomato, *Urtica*, and maize.^12,28,36,44^ These groups may have emerged after the divergence of monocotyledons and dicotyledons, and they play a unique role in monocotyledons or dicotyledons.

In previous studies, some scholars have suggested that SAURs play distinct molecular roles in different cellular compartments.^45^ For example, in the plasma membrane, SAURs interact with PP2C.D2/5/6 to inhibit the dephosphorylation of H±ATPases AHA1/2, leading to cell elongation.^46^ In the cytoplasm, *SAURs* may interact with SSPP (PP2C.D1) to inhibit the dephosphorylation of AtSARK, inducing senescence, such as in the case of OsSAUR39.^24^ The function of SAURs in the nucleus remains unclear, but they may interact with PP2C.D1/3/4 in the nucleus or bind to promoter regions as transcription factors to regulate growth and development, as seen in SAUR76 and MeSAUR1.^23,47^ Regarding SAURs located in mitochondria and chloroplasts, Chen^38^ hypothesized that they might be involved in the energy transfer process across two organelle membranes. In this study, approximately 40% of rice SAUR proteins were predicted to be located in the nucleus, approximately 44% in mitochondria and chloroplasts, and only 10% in the cell membrane and cytoplasm. The remaining proteins were predicted to localize from extracellular sources. This result is also reflected in maize, citrus, and cotton.^34,38,48^ Consequently, OsSAUR proteins play diverse roles in rice growth and development.

In addition, the prediction of cis-acting elements in the promoter region of *SAUR* gene is also an essential tool for studying the potential function of *SAUR* gene. These *SAURs*, as auxin-responsive genes, may be involved in auxin signal transduction pathways. Consequently, we predicted auxin-responsive cis-elements in the promoter region of *SAUR* genes in rice. The results revealed that most of the genes possessed auxin-responsive cis-elements. Furthermore, we discovered that not all sister pairs of *OsSAUR* genes have consistent promoter sequences, which suggests that closely related genes may not share common regulatory characteristics (Figure 5). This phenomenon is also observed in maize, but differs from rice, as studies have shown that the upstream flanking regions of sister pairs are highly similar.^31,38^ We infer that this inconsistency may be attributed to analytical tools. In addition to auxin, the expression of different sets of *SAUR* genes can be positively or negatively regulated by various abiotic stresses, such as auxin, ABA, MeJA, SA, gibberellin, flavonoid biosynthesis, light, cold, and drought. This finding is consistent with previous research.^45^ There are also studies demonstrating that *ARF7* can directly bind to the promoter region of *SAUR* and asymmetrically activate the expression of *SAUR19* in the tropic response of plants,^27^ and *SAUR63* promoter regulates GA-dependent stamen filament elongation.^49^ Regardlessly, the cis-element analysis indicates that *OsSAUR* genes can respond to various abiotic stresses.

To gain a better understanding of the potential function of *OsSAURs*, we examined their expression patterns using the RiceXPro database.^50^ The analysis revealed variations in the expression of *OsSAURs* across different tissues and in response to IAA treatment. For instance, *AtSAUR63* has been found to promote hypocotyl and staminal filament elongation.^16^ Furthermore, *OsSAURs* displayed diverse expression patterns in response to auxin, including up-regulation, down-regulation, and no response (Figure 6b). In citrus, 23 *CitSAURs* were found to respond to IAA treatment, with significant changes observed in the expression levels of 14 members in the AZ of the fruit.^48^ After applying IAA treatment to cotton, 11 out of 16 *GhSAUR* genes were up-regulated, while three were down-regulated.^34^ In a previous study, four *PavSAUR* genes (*PavSAUR13/16/55/61*) were found to be differentially expressed in the carpopodium and small fruits of the abscission zone.^28^ However, our treatment of 14-day-old rice with IAA did not align with the predicted transcriptome expression when we analyzed the *OsSAUR5/13/24/32/53* genes using qRT-PCR (Figure 7). This suggests that the replicated genes may not share common regulatory characteristics.^38^ We plan to perform transcriptome sequencing to further investigate the expression of *OsSAUR* genes, a phenomenon that has previously been reported in studies.^4,13,34,42^ However, it is surprising to note that not all promoter regions of *OsSAUR* genes with high expression contain auxin-responsive cis-elements, such as *OsSAUR5*, *OsSAUR19*, *OsSAUR37*, and *OsSAUR57*. In other words, the presence of auxin-responsive cis-elements does not necessarily indicate that a gene is induced by auxin. We speculate that these genes may have unannotated auxin-responsive cis-elements or participate in other regulatory networks related to auxin response. In summary, the expression patterns of different *OsSAUR* genes are highly diverse.

In summary, this article identifies a total of 60 *OsSAURs* and classifies them into 7 groups based on their phylogenetic relationship with *Arabidopsis*. Subsequently, the gene structure, phylogeny, chromosome location, gene duplication, and cis-elements of auxin response of *OsSAUR* genes were analyzed to determine their characteristics and potential functions. Microarray analysis showed that the expression patterns of *OsSAUR* gene family members are significantly different, indicating their functional diversity. This study provides new insights into the *SAUR* family of *rice* and lays the foundation for further research on its functional mechanisms.

## Supplementary Material

Supplementary Chart.doc

## Data Availability

All data generated or analyzed during this study were included in this published article and the additional files.

## Abbreviations

SAUR: SMALL AUXIN UP RNA
Aux/IAA: Auxin/Indole-3-Acetic Acid
GH3: Gretchen Hagen 3
ACS: Acid Synthase
GH2/4-like: Glutathione-S-transferase
BR: Brassinosteroids
GA: Gibberellic Acid
JA: Jasmonic Acid
ABA: Abscisic Acid
RGAP: Genome Databases
HMM: Hidden Markov Mode
NCBI: National Center for Biotechnology Information
NJ: Neighbor-joining
MEGA: Molecular Evolutionary Genetics Analysis
IAA: 3-Indoleacetic Acid
cDNA: Complementary DNA
ABRE: Abscisic Acid Responsive Elements
P-box: Permutation-box
GARE: Gibberellic Acid Responsive Elements
MBS: MYB-binding Elements
MeJA: Methyl Jasmonate
SA: Salicylic Acid
