# Supplementary material for: Genome-wide identification and expression analysis of SMALL AUXIN UP RNA (SAUR) genes in rice (Oryza sativa)
Source: Plant Signal Behav. 2024 Aug 15;19(1):2391658. doi: 10.1080/15592324.2024.2391658 (PMC11328882; doi:10.1080/15592324.2024.2391658)
Supplement: Supplementary Chart.doc [file KPSB_A_2391658_SM0068.doc]

| Prime names | Sequence (5′- 3′) |
| --- | --- |
| F-OsSAUR5 | AAGGAGGCAGGGCAAGCAGCAACAAG |
| R-OsSAUR5 | AGATACTCTGTGGGGATGACAAACCT |
| F-OsSAUR13 | ATGGGATGTTTTGTTCGGTTCGC |
| R-OsSAUR13 | AAGGTACACCAATGGCACCTCGA |
| F-OsSAUR24 | GCTCCCCTGCGACGAGGACCACTTC |
| R-OsSAUR24 | AGACGAGGCTGTCCATCCCTTGAAGCA |
| F-OsSAUR32 | CTTCACCTGGCTGGGAAGGG |
| R-OsSAUR32 | CGCAGTGGTCGAAGCCGTA |
| F-OsSAUR53 | TGAGGTGGAGAAAGCATTGCT |
| R-OsSAUR53 | AGTTGGCACCATAGAACCAGTGT |

Table S1 Primers for fluorescence quantification used in the Fig7

Table S2 cis-regulatory elements analysis of *OsSAUR* genes using PlantCARE database

| GENE Name | Auxin | | MeJA | ABA | SA | GA_3_ | | | Flavonoid biosynthetic | Light | Cold | Drought |
| --- | --- | --- | --- | --- | --- | --- | --- | --- | --- | --- | --- | --- |
|  | TGA-element | AuxRE-core | TGACG- motif | ABRE | TCA-element | P-box | GARE-motif | TATC-box | MBSI | G-box | LTR | MBS |
| OsSAUR1 | 0 | 0 | 0 | 3 | 2 | 1 | 0 | 0 | 0 | 4 | 2 | 2 |
| OsSAUR2 | 1 | 0 | 8 | 8 | 0 | 0 | 0 | 0 | 0 | 6 | 2 | 1 |
| OsSAUR3 | 2 | 0 | 2 | 0 | 1 | 1 | 0 | 0 | 1 | 0 | 0 | 0 |
| OsSAUR4 | 0 | 1 | 2 | 2 | 1 | 0 | 1 | 0 | 0 | 2 | 0 | 0 |
| OsSAUR5 | 0 | 0 | 2 | 4 | 0 | 1 | 0 | 0 | 0 | 3 | 0 | 2 |
| OsSAUR6 | 0 | 0 | 0 | 7 | 0 | 0 | 0 | 0 | 0 | 8 | 1 | 1 |
| OsSAUR7 | 1 | 0 | 2 | 1 | 0 | 0 | 0 | 0 | 0 | 1 | 0 | 2 |
| OsSAUR8 | 1 | 0 | 2 | 2 | 2 | 1 | 0 | 0 | 0 | 3 | 0 | 4 |
| OsSAUR9 | 0 | 0 | 0 | 2 | 0 | 0 | 0 | 1 | 0 | 0 | 2 | 0 |
| OsSAUR10 | 0 | 0 | 2 | 1 | 1 | 0 | 0 | 0 | 0 | 3 | 0 | 0 |
| OsSAUR11 | 2 | 1 | 8 | 8 | 0 | 0 | 0 | 0 | 1 | 6 | 0 | 0 |
| OsSAUR12 | 1 | 0 | 6 | 1 | 0 | 0 | 2 | 0 | 0 | 2 | 3 | 2 |
| OsSAUR13 | 0 | 0 | 4 | 5 | 0 | 0 | 1 | 0 | 0 | 6 | 1 | 2 |
| OsSAUR14 | 0 | 0 | 2 | 0 | 0 | 0 | 0 | 0 | 0 | 0 | 0 | 0 |
| OsSAUR15 | 1 | 0 | 14 | 3 | 0 | 0 | 0 | 0 | 0 | 5 | 1 | 2 |
| OsSAUR16 | 1 | 0 | 4 | 7 | 1 | 0 | 1 | 0 | 0 | 6 | 2 | 1 |
| OsSAUR17 | 1 | 0 | 16 | 8 | 0 | 0 | 0 | 0 | 0 | 7 | 1 | 0 |
| OsSAUR18 | 1 | 1 | 6 | 2 | 0 | 0 | 0 | 0 | 0 | 2 | 0 | 1 |
| OsSAUR19 | 0 | 0 | 14 | 7 | 0 | 0 | 0 | 0 | 0 | 9 | 2 | 1 |
| OsSAUR20 | 0 | 0 | 0 | 5 | 2 | 0 | 1 | 0 | 0 | 5 | 1 | 0 |
| OsSAUR21 | 0 | 0 | 2 | 5 | 0 | 0 | 0 | 0 | 0 | 3 | 2 | 1 |
| OsSAUR22 | 3 | 0 | 10 | 5 | 3 | 1 | 0 | 0 | 0 | 6 | 0 | 2 |
| OsSAUR23 | 1 | 0 | 4 | 2 | 1 | 1 | 0 | 1 | 0 | 2 | 0 | 1 |
| OsSAUR24 | 1 | 0 | 4 | 3 | 0 | 1 | 0 | 0 | 0 | 3 | 1 | 0 |
| OsSAUR25 | 0 | 1 | 2 | 1 | 1 | 0 | 0 | 1 | 0 | 2 | 1 | 0 |
| OsSAUR26 | 1 | 0 | 2 | 6 | 0 | 1 | 0 | 1 | 1 | 6 | 2 | 2 |
| OsSAUR27 | 0 | 0 | 0 | 3 | 0 | 0 | 1 | 0 | 0 | 3 | 0 | 1 |
| OsSAUR28 | 0 | 1 | 0 | 2 | 1 | 0 | 0 | 0 | 0 | 3 | 2 | 2 |
| OsSAUR29 | 0 | 0 | 0 | 3 | 3 | 0 | 0 | 0 | 0 | 4 | 1 | 1 |
| OsSAUR30 | 2 | 0 | 2 | 4 | 0 | 1 | 0 | 0 | 0 | 5 | 0 | 0 |
| OsSAUR31 | 1 | 0 | 2 | 1 | 2 | 0 | 1 | 0 | 0 | 1 | 0 | 0 |
| OsSAUR32 | 1 | 1 | 2 | 4 | 0 | 0 | 1 | 0 | 0 | 4 | 0 | 0 |
| OsSAUR33 | 0 | 1 | 2 | 1 | 0 | 0 | 0 | 0 | 0 | 1 | 0 | 2 |
| OsSAUR34 | 0 | 0 | 6 | 2 | 0 | 0 | 1 | 0 | 0 | 2 | 0 | 4 |
| OsSAUR35 | 1 | 0 | 2 | 1 | 0 | 0 | 0 | 0 | 0 | 2 | 0 | 2 |
| OsSAUR36 | 2 | 0 | 4 | 0 | 0 | 0 | 0 | 0 | 0 | 1 | 0 | 1 |
| OsSAUR37 | 0 | 0 | 8 | 8 | 0 | 1 | 0 | 0 | 0 | 7 | 0 | 0 |
| OsSAUR38 | 0 | 0 | 0 | 6 | 1 | 0 | 1 | 0 | 0 | 3 | 0 | 0 |
| OsSAUR39 | 1 | 1 | 4 | 3 | 1 | 0 | 0 | 0 | 0 | 4 | 0 | 2 |
| OsSAUR40 | 2 | 0 | 2 | 0 | 0 | 1 | 0 | 0 | 0 | 1 | 2 | 0 |
| OsSAUR41 | 1 | 0 | 2 | 6 | 1 | 1 | 0 | 0 | 0 | 5 | 1 | 1 |
| OsSAUR42 | 1 | 0 | 6 | 0 | 1 | 0 | 0 | 0 | 0 | 0 | 0 | 0 |
| OsSAUR43 | 0 | 1 | 8 | 3 | 0 | 0 | 0 | 0 | 0 | 3 | 0 | 1 |
| OsSAUR45 | 1 | 1 | 0 | 3 | 1 | 0 | 1 | 0 | 0 | 3 | 0 | 0 |
| OsSAUR46 | 0 | 0 | 0 | 1 | 4 | 1 | 0 | 0 | 0 | 2 | 1 | 1 |
| OsSAUR47 | 0 | 1 | 2 | 0 | 1 | 0 | 0 | 0 | 0 | 1 | 1 | 4 |
| OsSAUR48 | 0 | 0 | 2 | 1 | 1 | 0 | 0 | 0 | 0 | 0 | 0 | 2 |
| OsSAUR49 | 0 | 1 | 6 | 0 | 0 | 1 | 0 | 0 | 0 | 0 | 0 | 3 |
| OsSAUR50 | 0 | 0 | 4 | 3 | 0 | 1 | 0 | 0 | 0 | 2 | 0 | 3 |
| OsSAUR52 | 0 | 1 | 8 | 3 | 1 | 1 | 0 | 0 | 0 | 2 | 1 | 0 |
| OsSAUR53 | 2 | 0 | 2 | 4 | 0 | 0 | 0 | 0 | 0 | 4 | 0 | 3 |
| OsSAUR54 | 2 | 1 | 0 | 1 | 0 | 0 | 1 | 0 | 0 | 1 | 0 | 4 |
| OsSAUR55 | 1 | 2 | 8 | 2 | 2 | 0 | 1 | 0 | 0 | 2 | 1 | 5 |
| OsSAUR56 | 0 | 0 | 8 | 5 | 1 | 0 | 1 | 0 | 0 | 2 | 0 | 1 |
| OsSAUR57 | 0 | 0 | 8 | 3 | 1 | 0 | 0 | 0 | 0 | 1 | 0 | 2 |
| OsSAUR58 | 2 | 0 | 6 | 10 | 0 | 0 | 0 | 0 | 1 | 10 | 1 | 0 |
| OsSAUR59 | 1 | 1 | 4 | 4 | 0 | 0 | 0 | 0 | 0 | 5 | 0 | 1 |
| OsSAUR60 | 0 | 0 | 8 | 6 | 1 | 0 | 0 | 0 | 0 | 4 | 1 | 2 |
